# Supplementary material for: A bispecific immunotweezer prevents soluble PrP oligomers and abolishes prion toxicity
Source: PLoS Pathog. 2018 Oct 1;14(10):e1007335. doi: 10.1371/journal.ppat.1007335 (PMC6181439; doi:10.1371/journal.ppat.1007335)
Supplement: S1 Text — (DOCX) [file ppat.1007335.s001.docx]

**S1 Text**

In order to establish if scPOM-bi can bind intermolecularly to two PrP molecules we used SPR to measure the dissociation constant, and thus avidity effects, with different dilutions of PrP. Intermolecular avidity, in fact, would be present if a single scPOM-bi antibody molecule could interact with two PrP molecules immobilized in close proximity on the SPR chip surface. Intermolecular avidity would not be present, instead, when the PrP molecules are not in close proximity (infinite dilution). By contrast, intramolecular avidity would be detected in both cases. Although it is not possible to measure the binding properties on a single PrP molecule, if the avidity of scPOM-bi changes at increasing dilution of immobilized PrP then intermolecular binding is relevant. Intramolecular avidity would instead not be affected by dilution.

We therefore tested three different antibody constructs: 1) scPOM1, which has only one antigen binding site and thus no avidity; 2) POM1-IgG, two antigen binding sites and intermolecular avidity that can only arise from intermolecular binding of the GD of two PrP molecules; 3) scPOM-bi.

In all three cases the association constant, measured as control, did not change with PrP dilution (S2A Fig.), as expected since association should not be affected by avidity and bivalent binding. The dissociation of scPOM1 was equally unaffected by dilution (S2B Fig.), since avidity cannot be present with a single antigen binding site. The dissociation of POM1-IgG, by contrast, became faster at increased PrP dilution, as expected for loss of intermolecular avidity. This is because it is less likely to find two immobilized PrP molecules in close proximity at increasing dilution, leading to loss of intermolecular binding of POM1-IgG to two GD domains. The dissociation constant of scPOM-bi behaved just like that of POM1-IgG at increasing dilutions, indicating that intermolecular binding of one scPOM-bi to two PrP molecules is, indeed, possible.
