# Supplementary figures and images for: A bispecific immunotweezer prevents soluble PrP oligomers and abolishes prion toxicity
Source: PLoS Pathog. 2018 Oct 1;14(10):e1007335. doi: 10.1371/journal.ppat.1007335 (PMC6181439; doi:10.1371/journal.ppat.1007335)

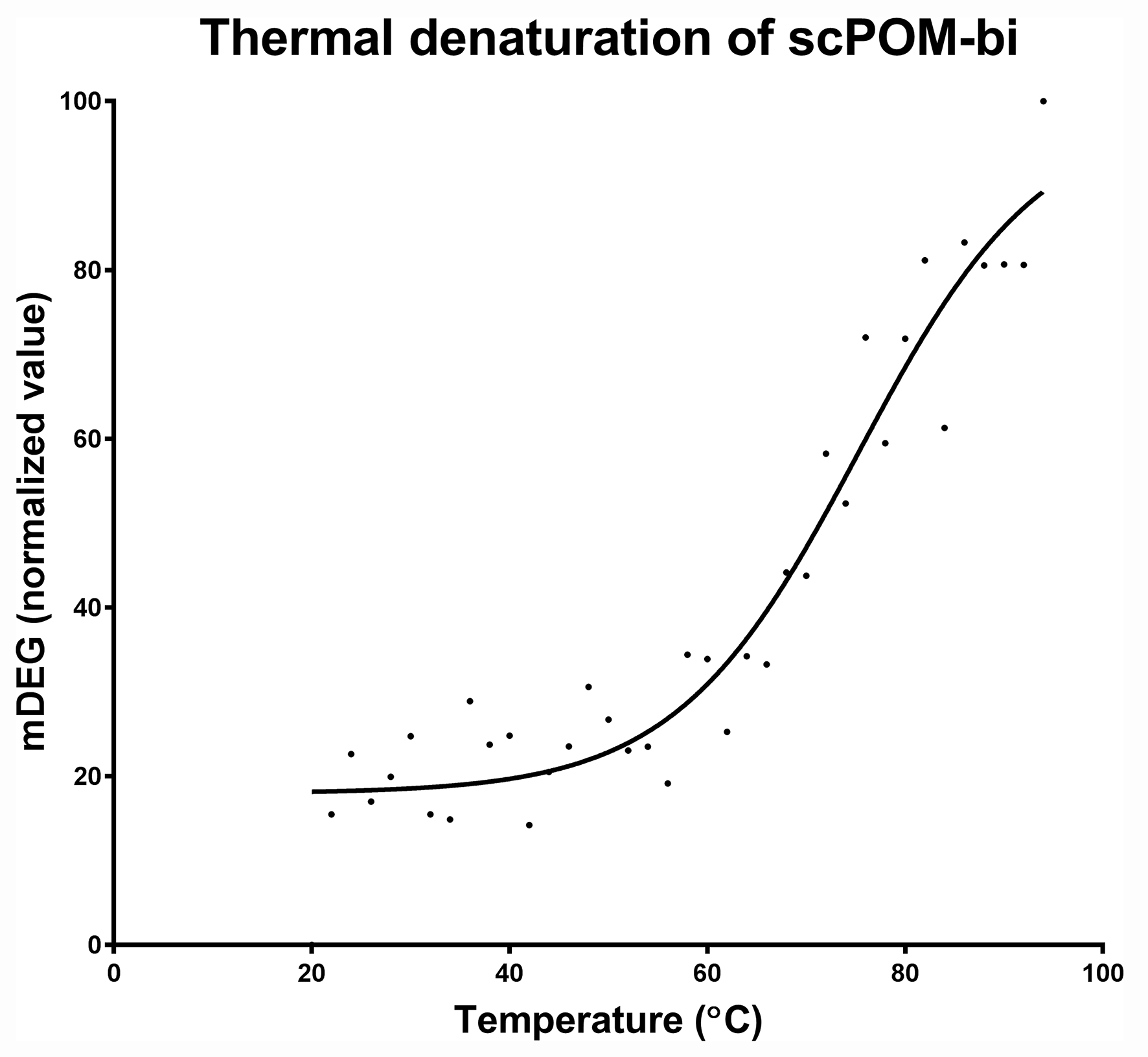

Supplement: S1 Fig — (TIF) [file ppat.1007335.s004.tif]

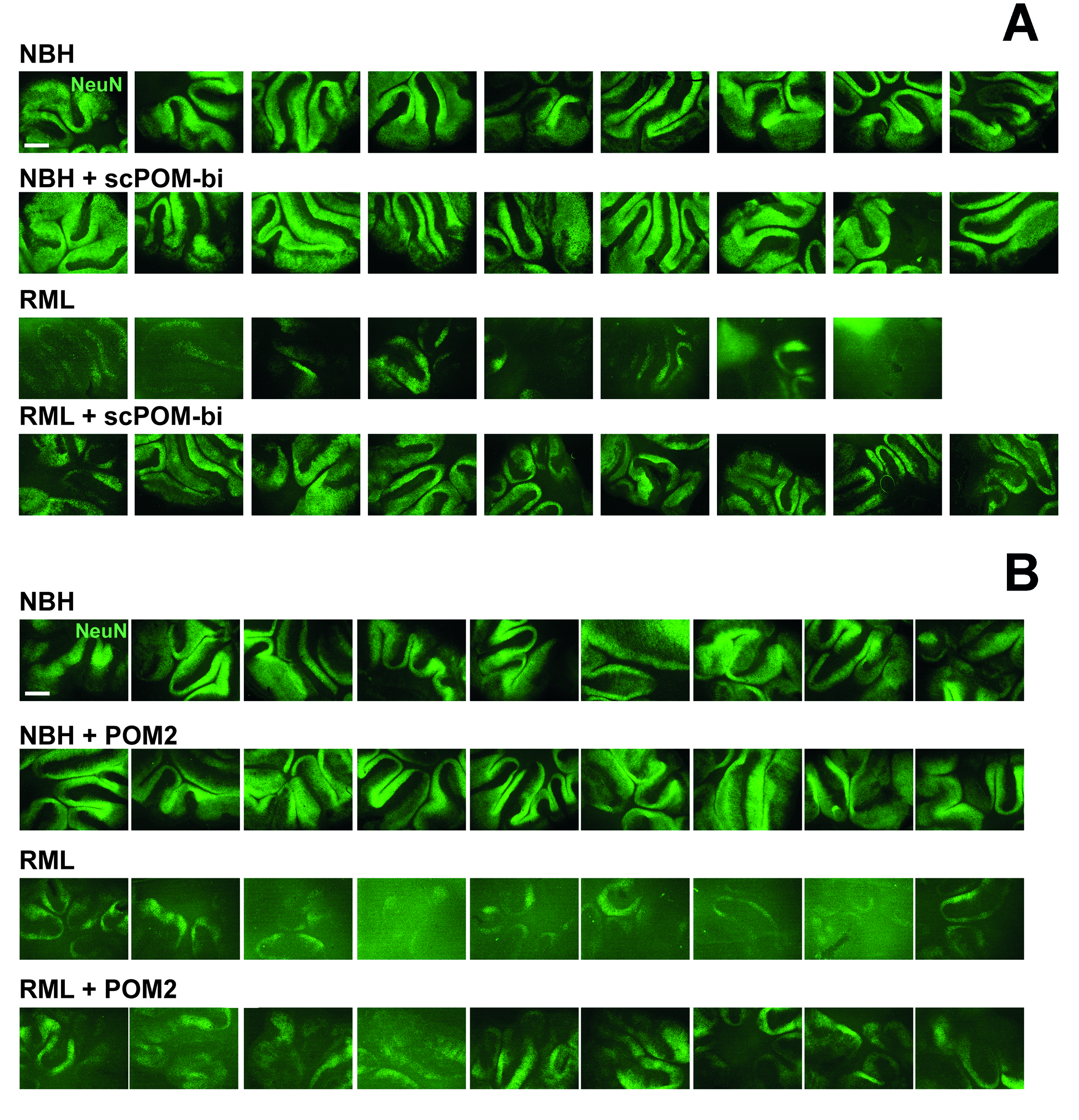

Supplement: S2 Fig — (A) scPOM-bi; (B) POM2 IgG. Scale bar = 500 μm. (TIF) [file ppat.1007335.s005.tif]

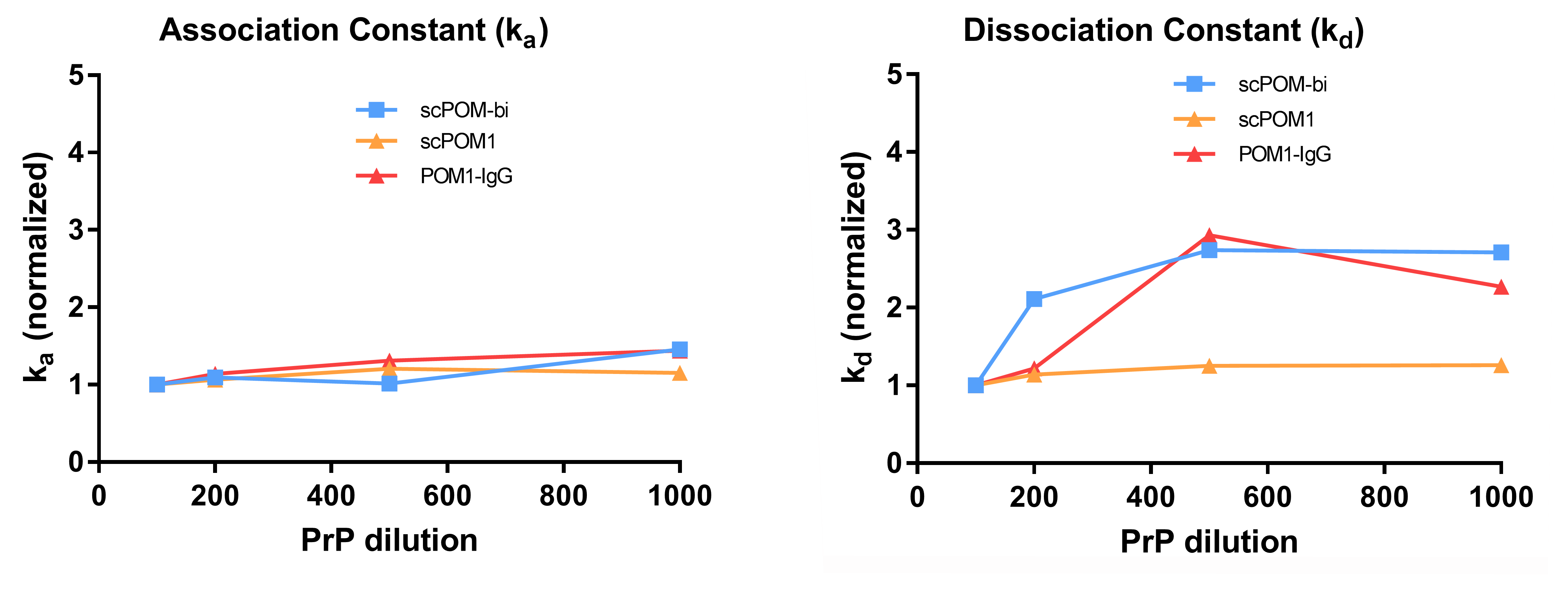

Supplement: S3 Fig — The dilution of PrP on the sensor chip is reported. The dissociation constant, but not the association, is affected by PrP dilution, indicating that intermolecular avidity effects are present in POM1 IgG and scPOM-bi. See S1 Text for further details. (TIF) [file ppat.1007335.s006.tif]

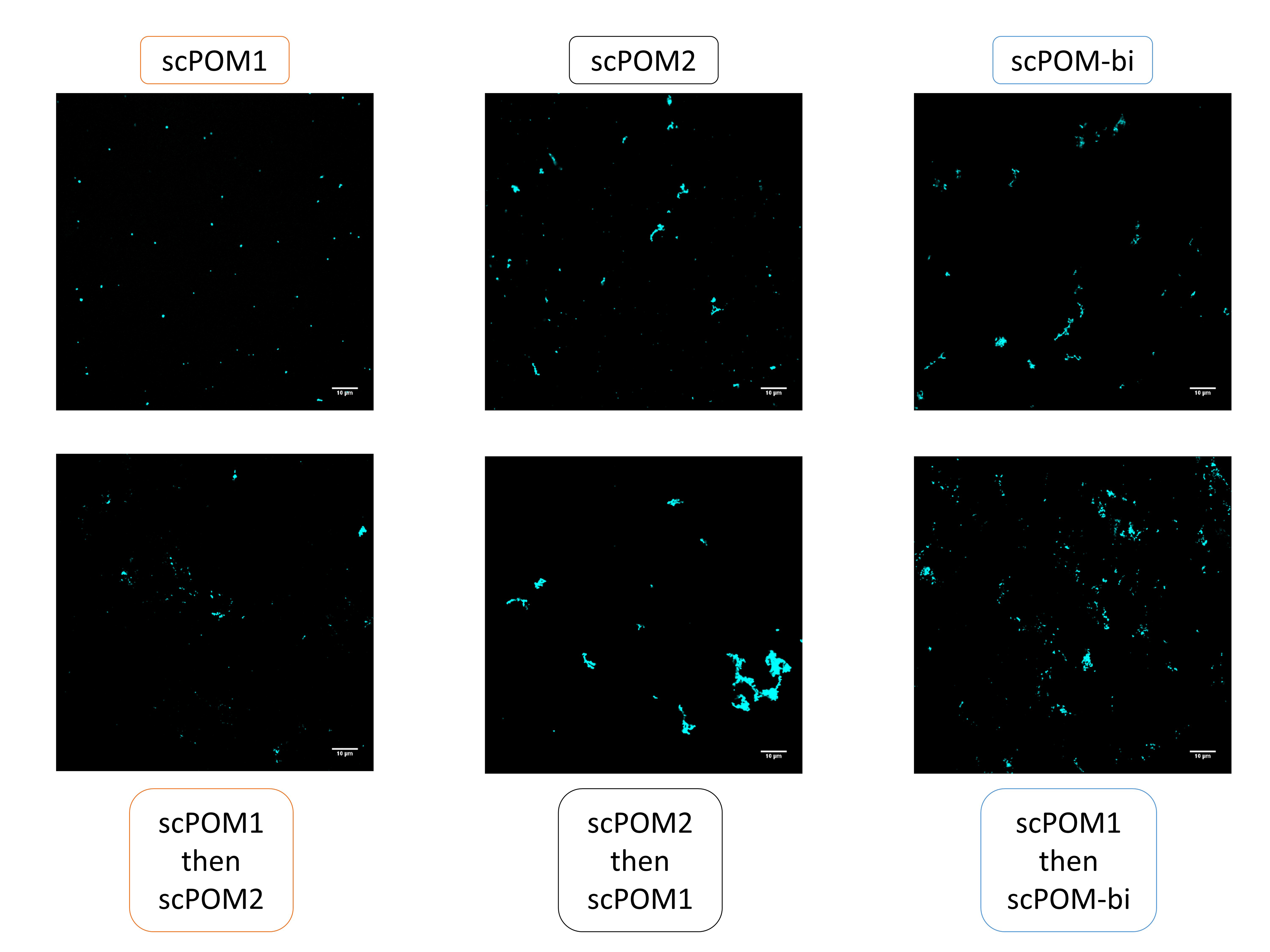

Supplement: S4 Fig — See main text (Fig 4) for quantification and methods for experimental details. Briefly, complexes between recombinant mPrP and antibodies were formed in vitro and the material deposited on microscopy slides without centrifugation or other purification steps. Species of different size are apparent when mPrP is in complex with toxic (POM1) or non toxic antibodies. (TIF) [file ppat.1007335.s007.tif]

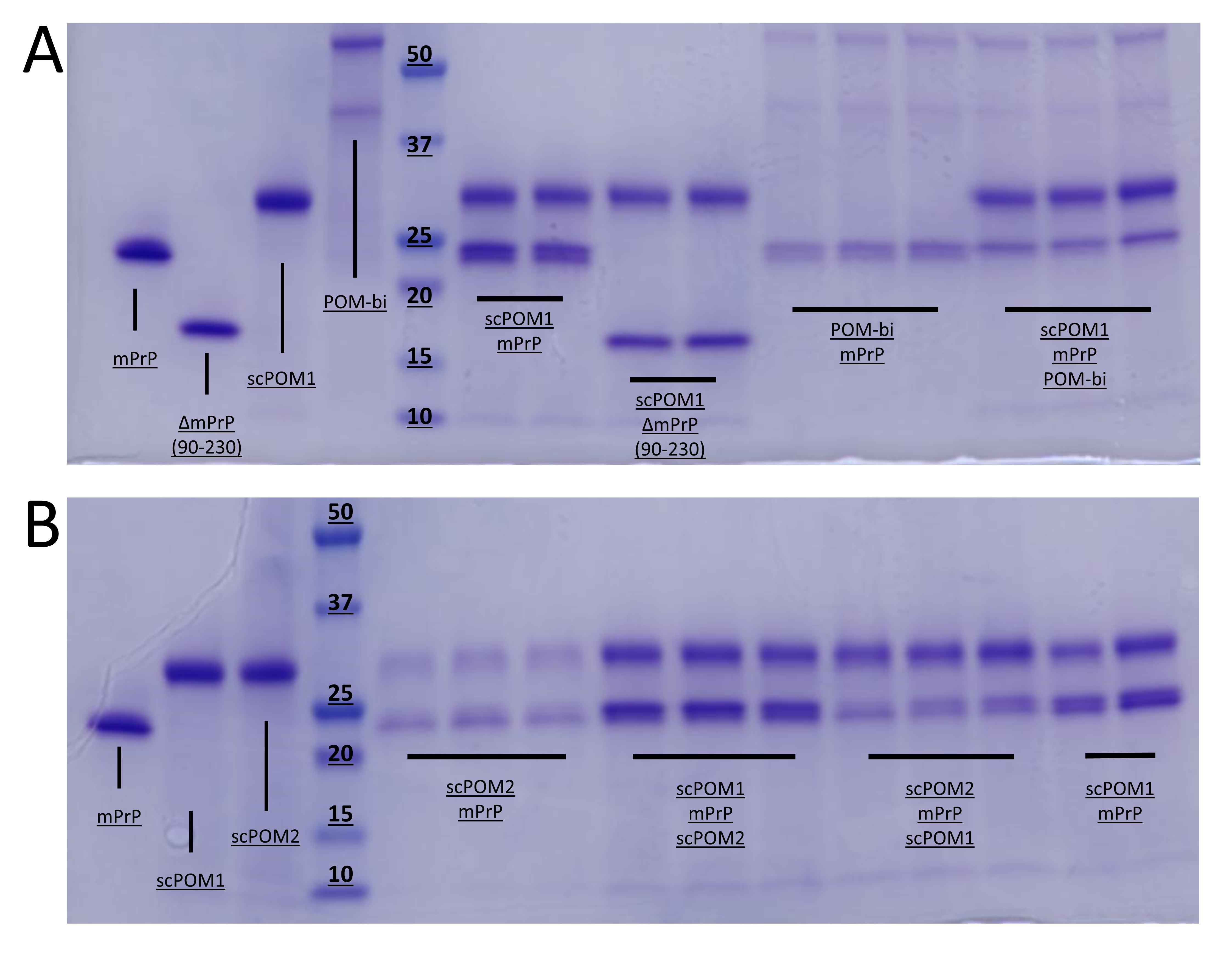

Supplement: S5 Fig — Precipitation assays confirmed that the toxic scPOM1:mPrP complex generates soluble oligomers containing both PrP and antibody (A). No soluble material was present in the complexes between mPrP and scPOM2 (B), scPOM-bi or when scPOM-bi was added after scPOM1 (A). See main text (Fig 4) for quantification and methods for experimental details. Briefly, after formation of the mPrP:Ab complexes in vitro the samples were centrifuged at 20’000 x g. The amount of mPrP and Ab present in the resulting supernatant was estimated with PAGE/Western Blot. The quantity of soluble material is reported as percentage of soluble mPrP or Ab alone, which do not precipitate or form aggregates over the observed time frame. The variability is due to the experimental set up and to the fact that we are analyzing transient, non-homogeneous species that are likely to change over time. Such variability does not affect the statistical significance of the measures. (TIF) [file ppat.1007335.s008.tif]

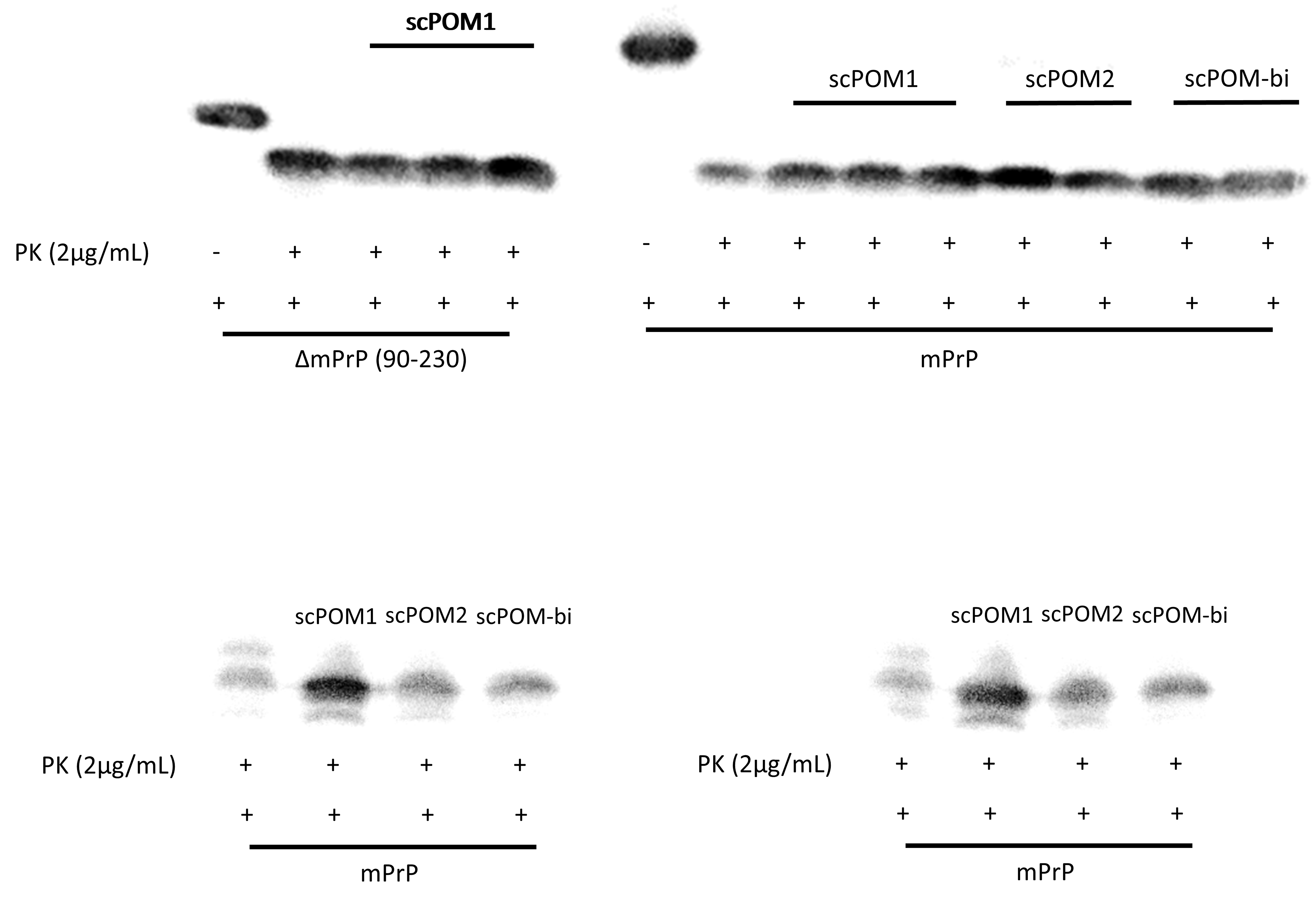

Supplement: S6 Fig — mPrP:Ab complexes were formed in vitro and 2μg/mL of Proteinase K were added. The presence of PK resistance species was assessed by western blot. An increased amount of species resistant to PK was detected in the scPOM1:mPrP complexes, but only if the flexible tail was present, which correlates to toxicity and protection assays. See main text (Fig 4) for quantification and statistics. (TIF) [file ppat.1007335.s009.tif]

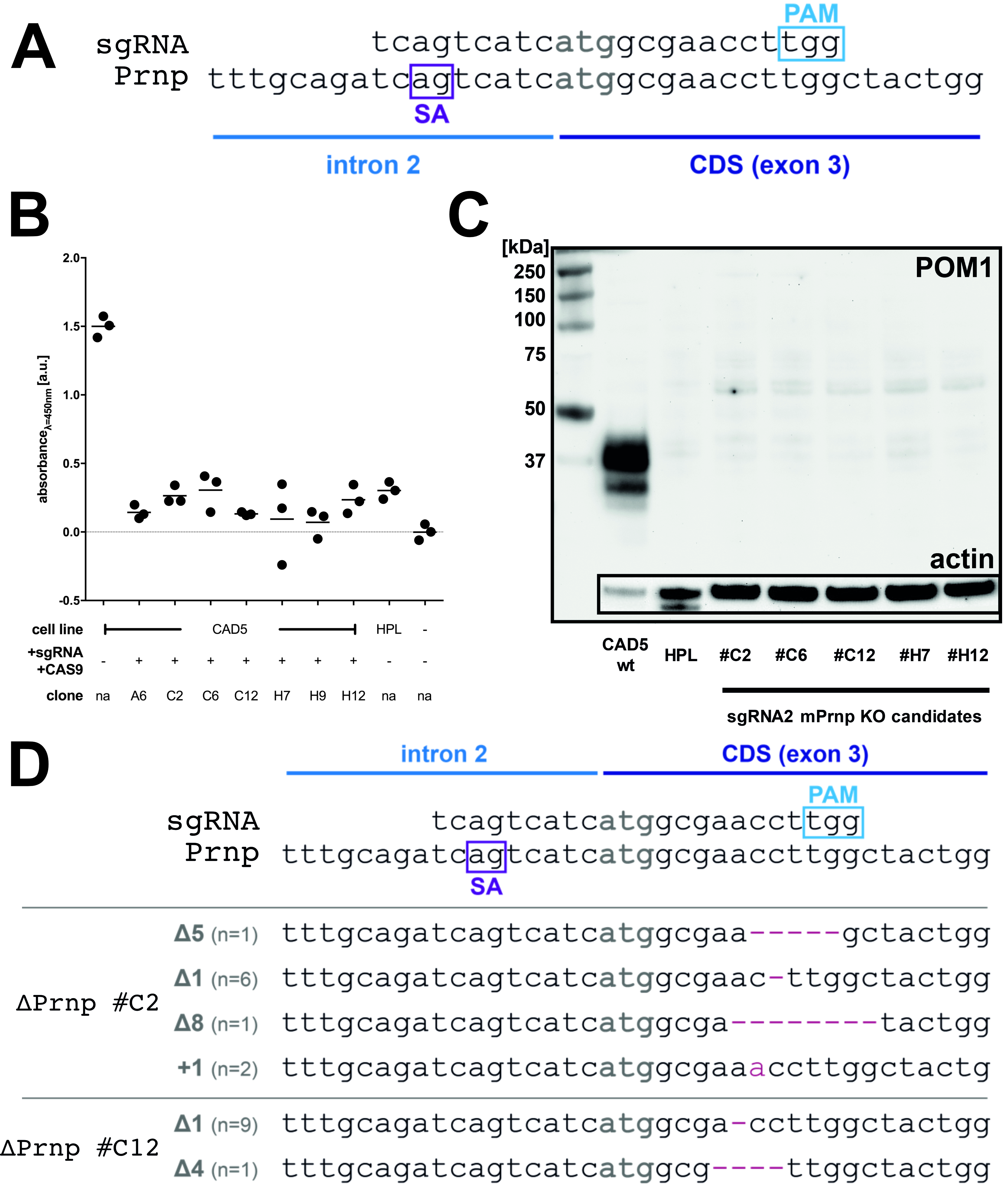

Supplement: S7 Fig — (A) Design of sgRNA for CRISPR/Cas9 mediated generation of CAD5 Prnp-/- cells. A PAM in the coding sequence of the signal peptide was chosen. (B) ELISA of 7 candidate CAD5 Prnp-/- clones showed similar PrPC levels compared to the established Prnp-/- cell line HPL (p>0.05, one-way ANOVA with Dunnett’s post-hoc test, all clones versus HPL), 5 of which were further assessed by PrPC western blot, confirming lack of PrPC expression (C). (D) Sanger sequencing of PCR amplified Prnp ORF showed n = 4 different mutations in #C2 and n = 2 different mutations, labelling according to (A). The splice acceptor site is unaffected in both of the constructs. (TIF) [file ppat.1007335.s010.tif]
